# The Occurrence of Gene Fusions in Thyroid Lesions and the Relation With Chronic Lymphocytic Thyroiditis

**DOI:** 10.1111/pin.70081

**Published:** 2026-01-05

**Authors:** Maaia Margo Jentus, Tom van Wezel, Dina Ruano, Marieke Snel, Abbey Schepers, Stijn Crobach, Hans Morreau

**Affiliations:** ^1^ Department of Pathology Leiden University Medical Center Leiden the Netherlands; ^2^ Department of Medicine Leiden University Medical Center, Division of Endocrinology Leiden the Netherlands; ^3^ Department of Surgery Leiden University Medical Center Leiden the Netherlands

**Keywords:** chronic lymphocytic thyroiditis, gene fusions, molecular diagnostics, molecular pathology, thyroid cancer

## Abstract

Previously, we concluded that thyroid resections with multifocal, genetically distinct lesions more often showed florid chronic lymphocytic thyroiditis (CLT) than thyroids with clonally related multiple lesions. In this study, we characterized a consecutive cohort of thyroid lesions for molecular drivers and investigated the relationship between the molecular alteration type and florid CLT. Molecular diagnostic data from 414 patients (2016–2025) were retrospectively reviewed, including clinical information and histopathological evaluation. Gene fusion, somatic mutation, and chromosomal LOH/imbalance/copy‐number analysis results were available for 342 cases. Eighty‐eight gene rearrangements were identified across 86 patients. Most had been previously reported in thyroid neoplasia. Five well‐known gene fusions revealed unusual breakpoints. Three gene fusions, previously reported only in nonthyroid malignancies (*BRAF–TRIM24*, *SLC12A7–TERT*, *PVT1–MYC*), were described for the first time in thyroid carcinoma. Three novel gene fusions (*TRIM65–RET*, *FGFR2–WARS1, PPARGC1A–PPARɣ)* produced in‐frame translation products leading to corresponding mRNA expression. *BRAF* exon‐skipping events were identified in treatment‐naïve papillary thyroid carcinomas. Florid CLT (*p* = 0.002) and younger age (OR = 0.97 per year, *p* < 0.001) were independently associated with gene fusion–positive tumors. Sex, follicular nodular disease, and Graves' disease were not significant predictors. Our findings suggest an association between fusion‐driven thyroid neoplasia and florid CLT, warranting further investigation.

## Graphical abstract

We identified both known and novel gene fusions in thyroid neoplasia, including several previously reported only in nonthyroid malignancies. Gene fusion–positive tumors were significantly (*p* < 0.001, annotated as ***) associated with florid chronic lymphocytic thyroiditis, unlike tumors driven by somatic mutations or copy number variations. Our findings expand the spectrum of molecular alterations in thyroid neoplasia and suggest a potential biological link between gene fusions and chronic inflammation.

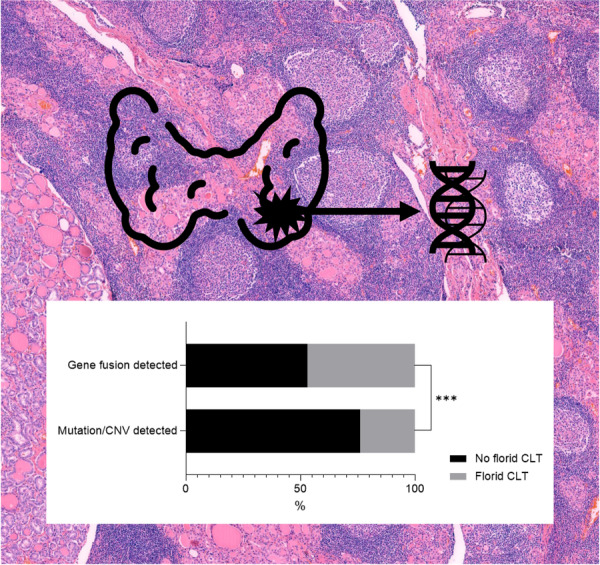

## 1 Introduction

Molecular testing is increasingly used across thyroid lesions, ranging from benign nodules to aggressive carcinomas. Thyroid cancer represents the most common endocrine malignancy [1]. While differentiated thyroid cancer generally has an excellent prognosis, even benign thyroid nodules cause a significant psychosocial burden [2]. Molecular testing can aid in the case of indeterminate fine‐needle aspiration (FNA) outcomes to prevent repeated FNAs or diagnostic hemithyroidectomies [3, 4].

The diverse molecular alterations can aid in the diagnostic classification and/or treatment choices [5]. Gene fusions are encountered in various thyroid tumors [6]. Some gene fusions are pathognomonic for a certain diagnosis, for example, *PAX8‐GLIS3* gene fusions are found in hyalinizing trabecular tumors [7]. *RET‐*, *NTRK‐*, or *ALK* gene fusions are found in classic or subtypes of papillary thyroid carcinomas (PTCs) [5]. The golden standards for gene fusions detection comprise random RNA (cDNA) sequencing, targeted NGS cDNA sequencing, or whole‐genome DNA sequencing. Optimal testing is able to detect novel gene partners of known genes or completely novel gene fusions. The elucidation of certain gene fusions can offer options for targeted therapies in case of advanced disease [8, 9]. Yet, the full spectrum of gene fusions in thyroid lesions might still be incomplete. In a recent study of patients with multifocal thyroid lesions, we concluded that cases with “multi‐unifocality/multiple synchronous primary tumors” (separate molecularly unique lesions) more common were accompanied by florid chronic lymphocytic thyroiditis (CLT), in contrast to cases with multifocal genetically identical tumors [10]. We therefore asked whether florid CLT occurs more frequently in tumors with gene fusions per se than in those without gene fusions.

## 2 Materials and Methods

The anonymized pathology reports of thyroid histology and cytology, including molecular data in the period from 2016 to 2025, were retrospectively reviewed. Pathology database queries selected reports containing the terms “fusion” and “thyroid” in the conclusion text. Most molecular reports included the testing performed on FNA specimens, with repeat testing on resection specimens when initial results were negative. The reported definitive classifying diagnosis was based on surgical resection report, and reviewed for WHO Classification of Endocrine Tumors, 5th Edition (2022) [11] compliance. Cases without subsequent surgical resection are explicitly indicated. After the first selection, a cohort of thyroid lesions that underwent anchored multiplex PCR‐based targeted NGS gene fusion analysis (https://www.palga.nl/voor-pathologen/moleculaire-bepaling see under “LUMC”) together with successful somatic mutation analysis was compiled. Cases with gene fusions detected by previously available methods (fluorescence in situ hybridization, gene fusion‐specific PCR, or NGS) and concurrent somatic mutation analysis were also included. This resulted in 342 cases with conclusive results. In all included cases, the presence of CLT was scored histologically. No clinical serological data were available.

Florid CLT was defined as dense, diffuse lymphocytic infiltration with or without secondary germinal centers. Peritumoral lymphocytic thyroiditis was defined as an infiltration around a neoplasm in the absence of infiltration in nonneoplastic tissue away of the tumor [12]. Focal LT was defined as focal small interfollicular lymphocytic aggregates without reactive follicular changes or germinal center formation. Peritumoral and focal LT were scored as absent. In the setting of Graves' disease, extensive lymphoplasmacytic infiltration with germinal center formation was scored as florid CLT when present.

According to the Central Committee on Research involving Human Subjects of our institute, this type of study does not require ethics approval in the Netherlands. The study was waived by the Medical Ethics Review Committee of the Leiden University Medical Center (G20.104). Data handling complied with the Code of Conduct for the Use of Data in Health Research (Federation of Dutch Medical Scientific Societies). Basic patient characteristics included sex and age. Lesion characteristics included CLT status, number of lesions, size, classifying diagnoses, and detected molecular alterations. Molecular workup, including DNA NGS panels and gene fusions panel used (Supporting Information S3: Supplemental Material, updates can be found at https://www.palga.nl/voor-pathologen/moleculaire-bepaling under “LUMC”) was described previously [13].

Descriptive statistics were used for baseline characteristics. Analyses were performed using Prism 10.2.3 (GraphPad Software Inc.) and IBM SPSS Statistics 29.0.0.0 (241). Quantitative parameters are presented with minimum, maximum, median, mean, and standard deviation (SD). Normality was assessed using the Kolmogorov–Smirnov and Shapiro–Wilk tests. Group comparisons used unpaired *t*‐tests (normally distributed data), Mann–Whitney *U* tests (non‐normal data), or one‐way ANOVA (multiple groups). Categorical variables were analyzed using *χ* ^2^ or Fisher's exact tests. A multivariate binary logistic regression was performed on all resected cases with available histological evaluation of CLT and complete data (*n* = 267, excluding cytology‐only cases), to assess whether the association between florid CLT was independently associated with gene‐fusion positivity. The dependent variable was gene fusion status (fusion‐positive vs. fusion‐negative). Covariates included age, sex, florid CLT, Graves' disease, and follicular nodular disease (FND). Odds ratios (OR) with 95% confidence intervals (CI) were calculated. A *p* < 0.05 was considered statistically significant.

## 3 Results

**Figure 1:**
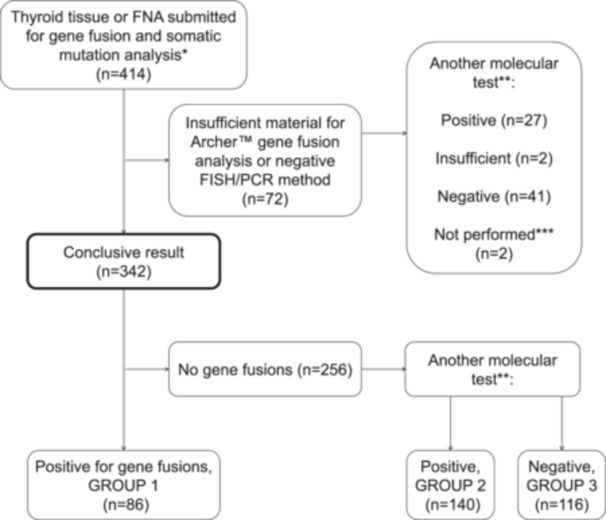
Flow diagram of case inclusion. A total of 414 reports were reviewed involving thyroid FNA or tissue specimens submitted for gene fusion and somatic mutation analysis. In total, 72 cases had insufficient material for reliable gene fusion analysis, after which 342 cases remained with conclusive results. Most were also analyzed for somatic mutations and copy number variations. CNV, copy number variation; FISH, fluorescence in situ hybridization; FNA, fine needle aspiration; IHC, immunohistochemistry; PCR, polymerase chain reaction. * Archer Fusion Plex, FISH, IHC, or fusion‐specific PCR. ** Somatic mutation analysis, pathogenic CNV analysis. *** Not performed due to unequivocally malignant histology.

Conclusive results of the gene fusion analysis were available in 342 patients (Figure 1). Gene fusions were detected in the samples of 86 patients (Group 1). Among the 256 gene‐fusion negative cases, 140 cases showed pathogenic DNA variants and/or copy number variations (CNV) (Group 2), and 116 were negative for both gene fusion analysis and pathogenic DNA alterations (Group 3).

**Figure 2:**
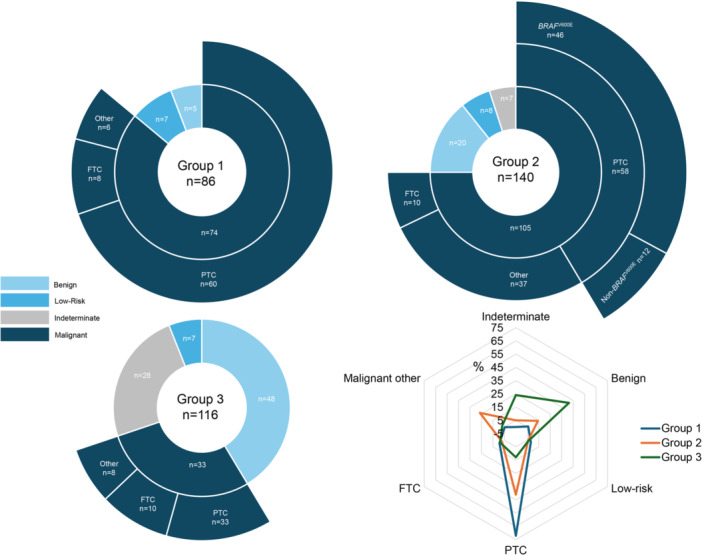
Diagnostic spectrum across the three molecular groups. Group 1 (*n* = 86) comprised all fusion‐positive cases; Group 2 (*n* = 140) included cases without gene fusion where pathogenic somatic mutation and/or copy number variation was detected; Group 3 (*n* = 116) had no detectable molecular alterations. In Group 3, 47 cases were benign upon resection (follicular nodular disease of the thyroid, follicular adenoma, or unassigned cell proliferations in the setting of chronic lymphocytic thyroiditis), 7 were low‐risk neoplasms (3 hyalinizing trabecular tumors and 4 noninvasive follicular thyroid neoplasms with papillary‐like nuclear features), 33 were malignant, and 27 remained indeterminate due to lack of surgical confirmation (BIII/BIV cytology), and 1 was a follicular tumor of uncertain malignant potential upon resection. FTC, follicular thyroid carcinoma; PTC, papillary thyroid carcinoma.

The three molecular groups encompassed lesions across the full diagnostic spectrum, from reactive and benign proliferations to low‐risk neoplasms and malignant tumors (Figure 2).

### 3.1 Group 1 (With Detected Gene Fusions)

**Figure 3:**
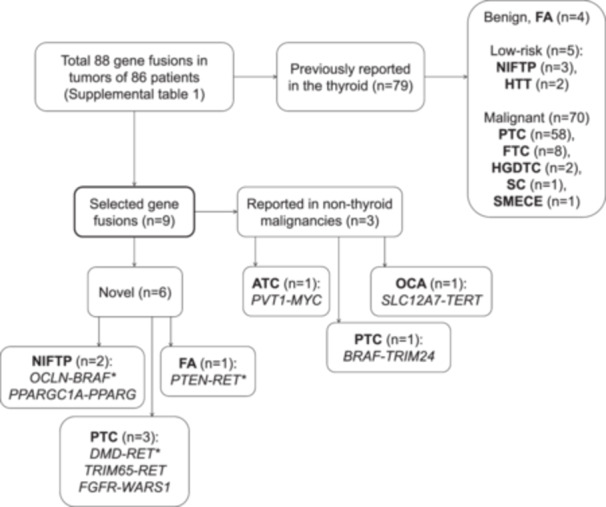
Overview of the 88 detected gene fusions. ATC, anaplastic thyroid carcinoma; FA, follicular adenoma; FTC, follicular thyroid carcinoma; HGDTC, high‐grade differentiated thyroid carcinoma; NIFTP, noninvasive follicular neoplasm with papillary‐like nuclear features; PTC, papillary thyroid carcinoma; SC, secretory carcinoma; SMECE, sclerosing mucoepidermoid carcinoma with eosinophilia. * No evident in_frame translation product.

In thyroid lesions of 86 patients, 88 gene fusions were detected (Figure 3, Supporting Information S2: Table 1). Five known gene fusions revealed unusual breakpoints. A high‐grade differentiated thyroid carcinoma (HGDTC, Patient 14) showed an *EML4* (NM_019063.4) Exon 5–*ALK* (NM_004304.4) Exon 20 gene fusion, instead of the common *EML4–ALK* V1–V3 variants, comprising Exons 13, 20, and 6 of *EML*, respectively, fused to Exon 20 of *ALK* [8, 14, 15]. This tumor also carried a *TERT* (NM_198253.2):c.−124C > T promoter mutation. An encapsulated classic PTC (Patient 181) showed an *EML4* Exon 6–*ALK* Exon 6 gene fusion. A PTC not otherwise specified (NOS) (Patient 142) revealed a *CCDC6* (NM_005436.4) Exon 1–*RET* (NM_020630.4) Exon 11 gene fusion. Two tumors (an invasive encapsulated follicular variant of PTC (IEFVPTC) of Patient 5 and an oncocytic PTC of Patient 7) showed an *NCOA4* (NM_005437.3) Exon 6–*RET* (NM_020630.4) Exon 12 gene fusion.

Not all detected gene fusions have been described in thyroid tumors. PTC NOS (Patient 347) harbored *BRAF* (NM_004333.4)–*TRIM24* (NM_003852.3) gene fusion, known from pancreatic adenocarcinoma [16], together with the reciprocal *TRIM24*–*BRAF* gene fusion reported in PTC [17]. A *SLC12A7* (NM_006598.2)–*TERT* (NM_198253.2) gene fusion, described in hepatocellular carcinoma [18], was detected in an oncocytic thyroid carcinoma (Patient 362), which also carried a pathogenic *KRAS* NM_004985.5:c.35 G > T, p.(Gly12Val) variant, and extensive chromosomal imbalances, but not the near‐homozygous genome pattern typical of oncocytic carcinoma [11, 19]. One anaplastic thyroid carcinoma (Patient 405) showed a *PVT1* (NR_003367.3)–*MYC* (NM_002467.6) gene fusion, previously reported in CRC [20] and medulloblastoma [21]. The underlying biology was described by Jin et al. [22].

Two treatment naïve PTCs showed *BRAF* large internal deletions/exon skipping events usually associated with acquired resistance to *BRAF* ^V600E^‐targeted therapy [23]: a noninvasive EFVPTC with high‐grade features [11] (Patient 89) with *BRAF* Exons 2–10 skipping, and an IEFVPTC (Patient 394) with *BRAF* Exons 4–10 skipping.

Three novel in‐frame gene rearrangements with predicted functional product were identified (*TRIM65*–*RET*, *FGFR2*–*WARS1, PPARGC1A*–*PPARɣ*). For each fusion, we assessed whether the 5′ partner is normally expressed in thyroid tissue using Human Protein Atlas data [24] or previously reported as 5′ partner in thyroid neoplasia, and screened the literature for evidence of pathogenicity of the 3′ partner.

*TRIM65* (NM_173547.4) Exon 5–*RET* (NM_020630.5) Exon 12 gene fusion was detected in a classical PTC (Patient 110) with relatively high *RET* mRNA expression. The mitotically active encapsulated PTC with a predominant follicular growth pattern [11] (Patient 399) harbored an *FGFR2* (NM_000141.4) Exon 17–*WARS1* (NM_004184.4) Exon 3 gene fusion preserved *FGFR2* expression. The *PPARGC1A* (NM_013261.5) Exon 5–*PPARɣ* (NM_005037.5) Exon 2 gene fusion in a noninvasive follicular neoplasm with papillary‐like nuclear features (NIFTP) (Patient 107) showed relative *PPARɣ* overexpression.

Several novel gene fusions lacked an apparent in_frame translation product (*PTEN‐RET*, *OCLN‐BRAF*, and *DMD‐RET)*. Although the fusions were qualitatively strong, no in_frame transcript could be inferred. However, for the *DMD* (NM_000109.3) Exon 49–*RET* (NM_020630.4) Exon 8 (Patient 127, solid/trabecular PTC) an unexpectedly high relative RET mRNA expression was revealed with fusion‐specific pattern, even though the Archer analysis predicted the gene fusion to be out of frame and manual sequence inspection supports this prediction. The *PTEN* (NM_000314.8) Exon 5–*RET* (NM_020630.5) Intron 10 gene fusion was detected in a follicular adenoma (Patient 74), a benign diagnosis was supported by absence of evident *RET* mRNA overexpression in the same assay. The *OCLN* (NM_002538.4) Exon 4–*BRAF* (NM_004333.6) Exon 10 gene fusion was found in a NIFTP (Patient 361).

### 3.2 Group 2 (No Detected Gene Fusions, Other Molecular Test Positive)

Among 140 patients of Group 2, 21 had a benign neoplasm (FA or OA), and 7 had indeterminate (*n* = 4) or unavailable postoperative diagnoses (BIV, *n* = 3). Eight tumors were diagnosed as NIFTP, all carrying *RAS* mutations (4x*NRAS*, 2x*KRAS, 2*x*HRAS)*. In total 105/140 patients had malignancy. Among 58 PTCs, 46 had *BRAF* ^V600E^. Other observed mutations were 5x*NRAS* (one with co‐occurring pathogenic splice variation in *EIF1AX)*, 2x*HRAS*, 1x*KRAS*, 1x*MAP2K1*, 1x*TERT* promoter (without another detected driver), and 1x*CDKN2B*. One cribriform morular variant of PTC harbored two APC mutations [25]. Among follicular carcinomas, *HRAS* mutations were most frequent (5 of 10). Of 20 oncocytic carcinomas, 14 showed extensive chromosomal losses; copy‐number analysis was not performed in 6.

### 3.3 Chronic Lymphocytic Thyroiditis

**Figure 4:**
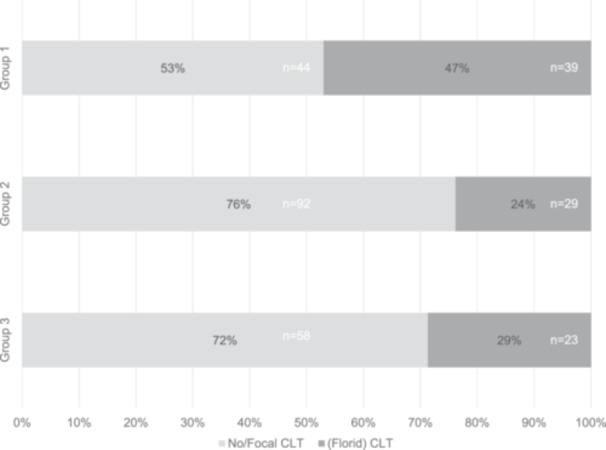
Frequency of florid chronic lymphocytic thyroiditis (CLT) in Group 1 (gene fusion‐positive), Group 2 (gene fusion‐negative with other molecular alterations), and Group 3 (negative for all molecular tests). Focal/peritumoral CLT was scored as absent. Florid CLT was significantly more frequent in Group 1 than in Group 2 (*p* < 0.001), and also more frequent in Group 1 than in Groups 2 and 3 combined (*p* = 0.0018). Distribution of diagnostic categories (benign, low‐risk, malignant) is shown in Supporting Information S1: Figure 1.

CLT status could be assessed in 83 specimens in Group 1, in 121 in Group 2, and in 81 in Group 3 (Figure 4).

Thyroids in Group 1 were significantly more likely to show florid CLT than those in Group 2 (*p* < 0.001). Among the 44 florid‐CLT‐negative patients in Group 1, 10 showed focal/peritumoral CLT. Of these 44 patients, 21 had underlying FND, 7 patients (of which 4 also had FND) had received prior chemotherapy for a nonthyroid malignancy, were immunosuppressed, or had a nonthyroid autoimmune disease. In six malignant cases, co‐occurring pathogenic somatic mutations or CNVs were found. No remarkable tumor‐related or clinical features were noted in the remaining 17 patients. However, 8 of these 17 patients were younger than 30 years old. Two patients in Group 1 had Graves' disease with extensive lymphoplasmacytic infiltration with germinal center formation, scored as florid CLT.

In Group 2, only 29 of 121 patients showed florid CLT, whereas 92 patients showed no (*n* = 67) or focal/peritumoral (*n* = 25) CLT. Among these 92 patients, 40 had FND, 3 had Graves' disease (2 concomitant with FND), 1 had diffuse hyperplasia compatible with Graves' disease but without clinical history, and 21 had a history a prior chemotherapy for a nonthyroid malignancy, immunosuppression, or a nonthyroid autoimmune disease. No relevant clinical history was identified in the remaining 40 patients, of whom only 2 were younger than 30 years.

Malignant lesions were analyzed separately. Florid CLT was significantly more frequent in Group 1 malignancies compared with Group 2 (49.3% vs. 22.2%; *p* < 0.001), both within PTC (50% vs. 29.6%; *p* = 0.036) and non‐PTC malignancies (45.5% vs. 11.1%; *p* = 0.02). Group 1 malignancies also showed a higher florid CLT frequency than Group 3 (49.3% vs. 19.4%, *p* = 0.005). Florid CLT frequencies did not differ between malignancies in Groups 2 and 3 (22.2% vs. 19.4%, *p* = 0.80), including within PTC (29.6% vs. 33.3%, *p* = 0.76) and non‐PTC malignancies (11.1% vs. 6.3%; *p* = 0.5).

Relationship between CLT status and age in Groups 1 and 2 is described in Supporting Information S4: Supplemental Text 1.

The relationship between FND and CLT was evaluated separately for each molecular group. In Group 1, florid CLT was significantly less frequent in tumors with a background of FND (27.6%) than in those without (57.4%; *p* = 0.009). No association was observed in Group 2 (*p* = 0.9). In Group 3, florid CLT was likewise less common in thyroids with FND (13.9% vs. 35.7%; *p* = 0.028). When all groups were combined, FND showed a significant negative association with florid CLT (*p* = 0.005).

In multivariate logistic regression including age, sex, florid CLT, Graves' disease, and FND, florid CLT remained independently associated with the presence of gene fusions (OR = 2.65, 95% CI 1.43–4.91, *p* = 0.002). Increasing age was inversely associated with gene fusion positivity (OR = 0.97 per year, *p* < 0.001). Sex, FND, and Graves' disease were not significant predictors (all *p* > 0.4).

## 4 Discussion

We analyzed molecular findings in thyroid pathology across a 9‐year period and examined their relationship with florid CLT and FND. Our data suggest a strong association between florid CLT and the presence of gene fusions.

In total, 88 gene fusions were identified in the tumors of 86 patients. Five PTCs showed unusual breakpoints in known gene fusions, and three gene fusions previously reported only in nonthyroid malignancies were detected in malignant thyroid tumors, two with additional somatic alterations.

The action mechanisms of established gene fusions and mutations are well characterized, whereas those of novel gene fusions still need to be investigated. Three novel in‐frame rearrangements showed support for functional relevance. *TRIM65*–*RET* (classic PTC) was associated with increased relative *RET* mRNA expression. An *FGFR2*–*WARS1* fusion (a mitotically active encapsulated PTC [11]) preserved *FGFR2* expression. While FGFR2‐fusions with C‐terminal truncations beyond Exons 17/18 lead to activation of FGFR2‐signaling [26, 27], this fusion may represent a double‐edged sword. *WARS1*‐fusions have been reported in breast cancer [28] and in SCC of the lung [28, 29]. The intact WARS1, which is ubiquitously [24] expressed is expected to have angiostatic activity and has been associated with favorable prognosis in colorectal cancer [30, 31]. Loss of function or truncation of WARS1 in the context of the fusion may therefore potentially have an effect on these protective functions.

A *PPARGC1A*–*PPARG* fusion (NIFTP) resulted in relative *PPARG* mRNA overexpression and likely encodes a product analogous to the well‐described *PAX8*–*PPARG* fusion as the fusion also starts from Exon 2 of PPARɣ [32, 33]. A subset of novel rearrangements appeared nonfunctional, lacking an in_frame translation product. *OCLN*‐*BRAF* (NIFTP) and *PTEN*‐*RET* (multifocal FA) gene fusions were qualitatively strong fusions, but no in‐frame translation product was extrapolated and there was absent *BRAF* or *RET* (over)expression, respectively. Although *DMD–RET* gene fusion (solid/trabecular PTC) was predicted to be out of frame, an unexpectedly high, fusion‐specific *RET* mRNA expression. This suggests that some out of frame rearrangements may still influence transcriptional activity, although the underlying mechanism remains unclear.

Previously, we concluded that thyroid resections with multifocal, genetically distinct lesions more often showed florid CLT than thyroids with clonally related multiple lesions [10]. We now show that florid CLT is significantly more common in thyroids with fusion‐positive tumors than in those driven by point mutations or with no detectable driver. This relationship remained significant in multivariate logistic regression after adjustment for age, sex, Graves' disease, and FND, supporting an independent association between thyroidal inflammation and fusion‐positive neoplasia.

Chronic inflammation is known to increase the risk of neoplastic transformation and can promote genetic instability [34, 35, 36, 37, 38]. In prostate cancer, inflammation has been linked to gene fusion formation. Similar mechanisms may plausibly contribute to gene fusion formation in the thyroid. While there is an established link between florid CLT and thyroid cancer [39, 40], patients with CLT may also have increased nonthyroid cancer risk [29].

Our results are consistent with previous observations on the occurrence of gene fusions in thyroid neoplasia. Patients with fusion‐positive tumors were significantly younger than those with tumors showing other or no molecular alterations, independent of CLT status. Within our cohort, no additional factors, such as prior chemotherapy for another malignancy, immunosuppression, or nonthyroid autoimmune disease, showed any association with fusion status. Fusion‐positive PTCs are known to occur more frequently in younger individuals [41, 42], a phenomenon partly attributed to prior irradiation [43], and underlying defects in DNA double‐strand repair pathways (ATM–CHEK2–BRCA) in pediatric and early‐onset thyroid cancer [44, 45].

In conclusion, our study documents known and novel gene fusions in thyroid neoplasia, including several rearrangements previously exclusively reported in nonthyroid neoplasia. Fusion‐positive neoplasms showed a stronger association with the presence of florid CLT than mutation‐driven or driver‐negative tumors, even after adjustment for clinical covariates. This finding requires validation in future studies.

### 4.1 Limitations

This study is retrospective and pathology‐based, and therefore clinical data such as thyroid function tests, autoantibodies, and ultrasound findings were not available. Histological assessment of CLT was the only indicator of thyroidal inflammation. Rare gene fusions were typically observed only once, limiting the ability to draw firm conclusions regarding fusion‐specific histological or clinical patterns. Protein‐level validation was not feasible due to limited resources. These factors should be taken into account when interpreting the findings.

## Author Contributions

Conceptualization: Maaia Margo Jentus and Hans Morreau. Methodology: Maaia Margo Jentus, Hans Morreau, Stijn Crobach, Tom van Wezel, and Dina Ruano. Formal analysis: Maaia Margo Jentus, Tom van Wezel, and Dina Ruano. Clinical resources: Marieke Snel and Abbey Schepers. Data curation: Maaia Margo Jentus. Writing – original draft preparation: Maaia Margo Jentus. Writing – review and editing: all authors. Visualization: Maaia Margo Jentus. All authors have read and agreed to the published version of the article.

## Funding

The authors received no specific funding for this work.

## Conflicts of Interest

The authors declare no conflicts of interest.

## Supporting information

Supplementary Figure 1.

Supplemental Table 1. Detected gene rearrangements.

Supplemental Material.

Supplemental Text 1.

## Data Availability

The data presented in this study are available from the corresponding author upon request.
