# Supplementary figures and images for: The Occurrence of Gene Fusions in Thyroid Lesions and the Relation With Chronic Lymphocytic Thyroiditis
Source: Pathol Int. 2026 Jan 5;76(1):e70081. doi: 10.1111/pin.70081 (PMC12835965; doi:10.1111/pin.70081)

## Slide 1
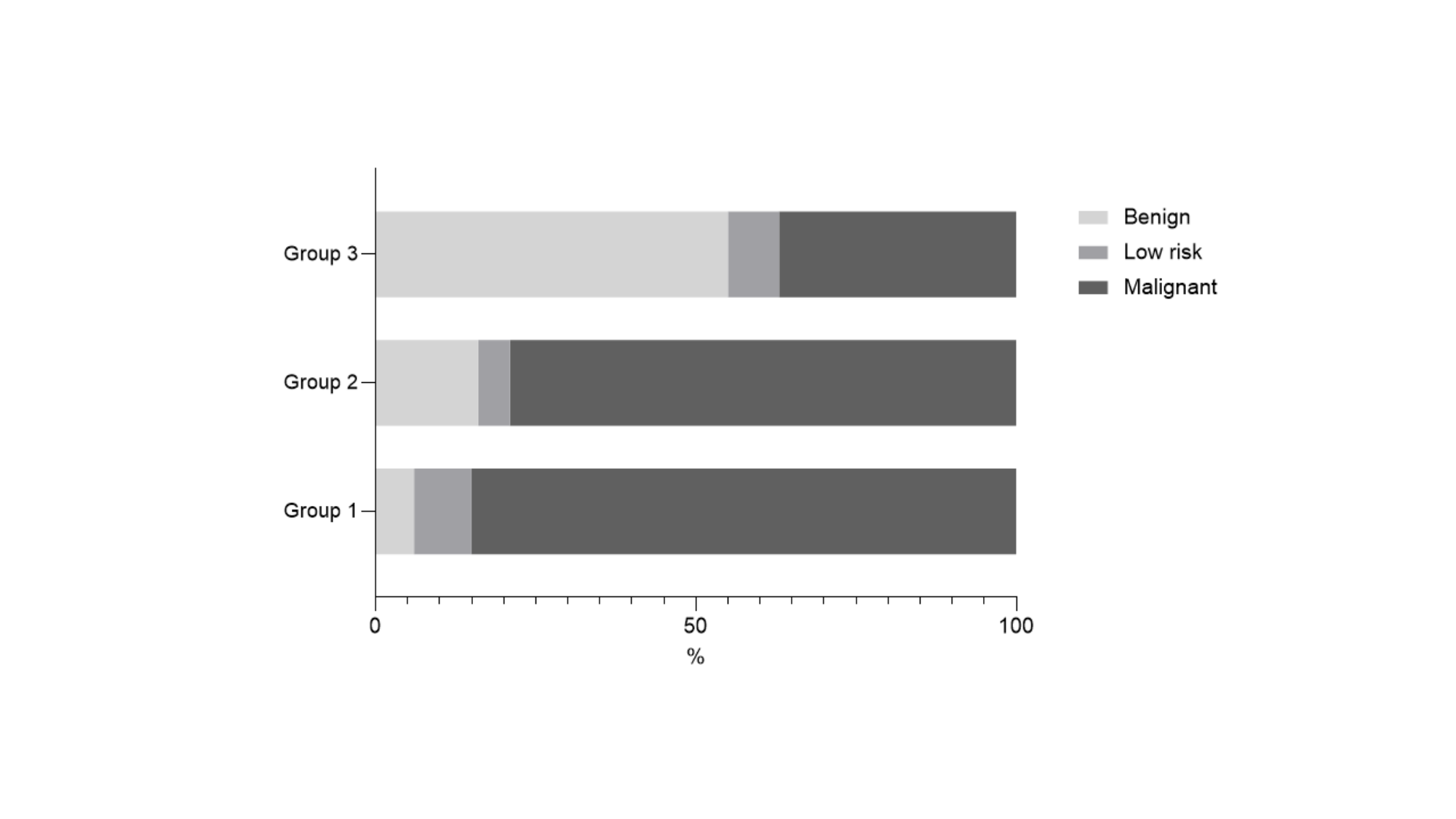

Supplement: Supplementary file 1 — Supplementary Figure 1. [file PIN-76-0-s002.pptx]
