# Supplementary material for: The Occurrence of Gene Fusions in Thyroid Lesions and the Relation With Chronic Lymphocytic Thyroiditis
Source: Pathol Int. 2026 Jan 5;76(1):e70081. doi: 10.1111/pin.70081 (PMC12835965; doi:10.1111/pin.70081)
Supplement: Supplementary file 2 — Supplemental Table 1. Detected gene rearrangements. [file PIN-76-0-s004.docx]

| **Supplemental table 1.** **Detected gene rearrangements.** | | | | | | | | | | |  |
| --- | --- | --- | --- | --- | --- | --- | --- | --- | --- | --- | --- |
| N | Age | Sex | Gene fusion | Gene fusion short | First reported by | Other molecular alterations | Diagnosis/  variant | Size (cm) | CLT | Details |  |
| **Benign** | | | | | | | | | | |  |
| 279 | 60 | f | *PAX8* (NM_00003466.3) exon 10 - *PPARɣ* (NM_005037.54) exon 2 | *PAX8-PPARɣ* | Marques et al.^1^ | nd | FA | 2.4 | + | FND |  |
| 221 | 40 | m | *PAX8* (NM_013952.3) exon 10 - *PPARɣ* (NM_005037.5) exon 2 | *PAX8-PPARɣ* | Marques et al.^1^ | nd | FA | 7 | no | FND |  |
| 243 | 67 | f | *PAX8* (NM_003466.3) exon 9 - *PPARɣ* (NM_005037.5) exon 2 | *PAX8-PPARɣ* | Marques et al.^1^ | nd | FA | 3 | no | FND; >10 years prior RT for breast cancer |  |
| 372 | 18 | f | *PAX8* (NM_003466.3) exon 9 - *PPARɣ* (NM_005037.5) exon 2 | *PAX8-PPARɣ* | Marques et al.^1^ | VUS *ERCC2* c.1802G>A, p.(Arg601Gln) | FA | 3 | no |  |  |
| 74 | 56 | m | *PTEN* (NM_000314.8) exon 5 - *RET* (NM_020630.5) intron 10 | *PTEN-RET* | Novel | nd | FA  (multifocal) | 5 | no | No *RET* expression. |  |
| **Low risk neoplasm** | | | | | | | | | | |  |
| 71 | 56 | f | *NCOA6* (NM_014071.3) exon 9 - *PPARɣ* (NM_005037.5) exon 2 | *NCOA6-PPARɣ* | Aydemirli and Morreau^2^ | nd | NIFTP | 3 | no | simultaneous 2 micro foci PTC (*KRAS* mutant) |  |
| 387 | 31 | f | *PAX8* (NM_003466.4) exon 2 - *GLIS3* (NM_152629.3) exon 1 | *PAX8-GLIS3* | Nikiforova et al.^3^ | nd | HTT | 5.2 | + |  |  |
| 402 | 71 | m | *PAX8* (NM_003466.4) exon 2 - *GLIS3* (NM_152629.3) exon 2 | *PAX8-GLIS3* | Nikiforova et al.^3^ | nd | HTT | 0.35 | + | simultaneous FVPTC |  |
| 67 | 57 | f | *PAX8* (NM_00466) exon 2 - *PPARɣ* (NM_005037) exon 2 | *PAX8-PPARɣ* | Brandler et al.^4^ | nd | NIFTP | 1.5 | no | FND |  |
| 229 | 49 | f | *PAX8* (NM_003466.3) exon 2 - *PPARɣ* (NM_005037.5) exon 2 | *PAX8-PPARɣ* | Brandler et al.^4^ | nd | NIFTP | 1.5 | no | FND;  simultaneous FA (*NRAS* mutant) and FA (no molecular analysis) |  |
| 107 | 37 | f | *PPARGC1A (*NM_013261.5) exon 5 - *PPARɣ* (NM_005037.5) exon 2 | *PPARGC1A-PPARɣ* | Novel | nd | NIFTP | 8 | no | *PPARɣ* overexpression |  |
| 361 | 53 | f | *OCLN* (NM_002538.4) exon 4 - *BRAF* (NM_004333.6) exon 10 | *OCLN-BRAF* | Novel | nd | NIFTP | 0,6 | + | No in frame translation product.  FND; simultaneous *BRAF*^V600E^ PTC, and FA. |  |
| **Malignant** | | | | | | | | | | |  |
| 89 | 67 | m | *BRAF* (NM_004333.6) exon 1 - *BRAF* (NM_004333.6) exon 11 | *BRAF* exon  2-10 skipping | Poulikakos et al.^5^ | Activating mutation in *TERT* c.-124C>A | Non-invasive EFVPTC with high-grade features^6^ | 9 | no | simultaneous NIFTP without fusion or *TERT*-mutation |  |
| 394 | 22 | m | *BRAF* (NM_004333.6) exon 3 - *BRAF* (NM_004333.6) exon 11 | *BRAF* exon  4-10 skipping | Poulikakos et al.^5^ | nd | IEFVPTC | 2,1 | no |  |  |
| 347 | 43 | f | *BRAF* (NM_004333.4) exon 10 - *TRIM24* (NM_003852.3) exon 3  *TRIM24* (NM_003852.3 exon 2 - *BRAF* (NM_004333.4) exon 11 | *BRAF-TRIM24*  *TRIM24-BRAF* | Heinrich et al. in ductal PancAC^7^  Hang et al.^8^ | nd | PTC NOS | 0.6 | + | *FND*  *BRAF-TRIM24* previously has not been reported in thyroid |  |
| 11 | 18 | f | *CCDC6* (NM_005436.4) exon 8 – *RET* (NM_020630.4) exon 12 | *CCDC6-RET* | Fusco et al.^9^ | nd | classic PTC | 6.2 | + | metastatic |  |
| 28 | 15 | f | *CCDC6* (NM_005436.4) exon 8 – *RET* (NM_020630.4) exon 12 | *CCDC6-RET* | Fusco et al.^9^ | nd | PTC NOS | 2.9 | + | multifocal, metastatic |  |
| 36 | 21 | f | *CCDC6* (NM_005436.4) exon 1 – *RET* (NM_020630.4) exon 12 | *CCDC6-RET* | Fusco et al.^9^ | nd | PTC NOS | 1.2 | no | FND;  metastatic |  |
| 81 | 24 | f | *CCDC6* (NM_005436.4) exon 1 – *RET* (NM_020630.4) exon 12 | *CCDC6-RET* | Fusco et al.^9^ | nd | classic PTC | 1 | + | metastatic |  |
| 95 | 59 | m | *CCDC6* (NM_005436.4) exon 1 – *RET* (NM_020630.4) exon 12 | *CCDC6-RET* | Fusco et al.^9^ | nd | oncocytic PTC | 0.9 | focal | multifocal; history of therapy for non-seminoma testis |  |
| 180 | 41 | m | *CCDC6* (NM_005436.4) exon 1 – *RET* (NM_020630.4) exon 12 | *CCDC6-RET* | Fusco et al.^9^ | nd | infiltrative FVPTC | 1 | no | metastatic |  |
| 186 | 26 | f | *CCDC6* (NM_005436.4) exon 1 – *RET* (NM_020630.4) exon 12 | *CCDC6-RET* | Fusco et al.^9^ | nd | IEFVPTC | 1.8 | + | metastatic |  |
| 190 | 42 | f | *CCDC6* (NM_005436.4) exon 1 – *RET* (NM_020630.4) exon 12 | *CCDC6-RET* | Fusco et al.^9^ | nd | diffuse sclerosing PTC | 0.8 | + |  |  |
| 271 | 55 | f | *CCDC6* (NM_005436.4) exon 1 – *RET* (NM_020630.4) exon 12 | *CCDC6-RET* | Fusco et al.^9^ | nd | classic PTC | 9.5 | + | multifocal;  metastatic |  |
| 142 | 37 | f | *CCDC6* (NM_005436.4) exon 1 – *RET* (NM_020630.4) exon 11 | *CCDC6-RET* | This study* | nd | PTC NOS | 1.8 | + | FND;  simultaneous *BRAF*^V600E^ PTC |  |
| 16 | 56 | f | *CCDC6* (H4)/RET (RET/PTC1) via RT-PCR | *CCDC6-RET* | Fusco et al.^9^ | not tested | PTC NOS | 1 | + | simultaneous macrofollicular adenoma |  |
| 413 | 14 | f | *CCDC30* (NM_001080850.3) exon 11 – *ROS1* (NM_002944.2) exon 36 | *CCDC30-ROS1* | Ritterhouse et al.^10^ | nd | PTC NOS | 2.7 | na |  |  |
| 127 | 48 | f | *DMD* (NM_000109.3) exon 49 - *RET* (NM_020630.4) exon 8 | *DMD-RET* | Novel | nd | solid/trabecular PTC | 1.4 | + | no apparent in-frame translation product, high relative *RET* mRNA expression with a fusion specific pattern |  |
| 14 | 60 | m | *EML4* (NM_019063.4) exon 5 - *ALK* (NM_004304.4) exon 20 | *EML4-ALK* | This study* | *TERT*p c.-124C>T | HGDTC | 11 | no | metastatic |  |
| 181 | 15 | f | *EML4* (NM_019063.4) exon 6 - *ALK* (NM_004304.4) exon 6 | *EML4-ALK* | This study* | nd | encapsulated classic PTC | 3 | no | nodular and diffuse hyperplasia |  |
| 197 | 8 | f | *EML4* (NM_019063.4) exon 13 - *ALK* (NM_004304.4) exon 20 | *EML4-ALK* | Demeure et al.^11^ | nd | PTC NOS | 5 | no |  |  |
| 224 | 53 | f | *EML4* (NM_019063.4) exon 13 - *ALK* (NM_004304.4) exon 20 | *EML4-ALK* | Demeure et al.^11^ | nd | classic PTC | 0.6 | + | FND |  |
| 38 | 25 | f | *EML4* (NM_019063) exon 2 - *NTRK3* (NM_002530) exon 14 | *EML4-NTRK3* | Liang et al.^12^ | nd | classic PTC | 2 | focal | metastatic |  |
| 358 | 47 | f | *EML4* (NM_019063) exon 2 - *NTRK3* (NM_002530) exon 14 | *EML4-NTRK3* | Liang et al.^12^ | nd | classic PTC | 2 | + |  |  |
| 272 | 32 | f | *ERC1* (NM_178039.3) exon 15 - *RET* (NM_020630.4) exon 12 | *ERC1-RET* | Makata et al.^13^ | nd | PTC NOS | 7 | + | metastatic |  |
| 345 | 16 | m | *ERC1* (NM_178039.3) exon 12 - *RET* (NM_020630.4) exon 12 | *ERC1-RET* | Nakata et al.^13^ | nd | classic PTC | 1 | + | multifocal, metastatic |  |
| 9 | 16 | f | *ETV6* (NM_01987.4) exon 4 - *NTRK3* (NM_002530.3) exon 1 | *ETV6-NTRK3* | Ricarte-Filho et al.^14^ | nd | PTC NOS | 2.5 | + | multifocal |  |
| 27 | 18 | f | *ETV6* (NM_01987.4) exon 4 - *NTRK3* (NM_002530.3) exon 14 | *ETV6-NTRK3* | Ricarte-Filho et al.^14^ | nd | PTC variant^15^ | 4.2 | + | metastatic |  |
| 131 | 19 | f | *ETV6* (NM_01987.4) exon 4 - *NTRK3* (NM_002530.3) exon 13 | *ETV6-NTRK3* | Ricarte-Filho et al.^14^ | nd | classic PTC | 7 | na | metastatic;  simultaneous PTC contralateral |  |
| 135 | 73 | f | *ETV6* (NM_01987.4) exon 4 - *NTRK3* (NM_002530.3) exon 13 | *ETV6-NTRK3* | Ricarte-Filho et al.^14^ | nd | PTC variant^15^ | 3 | + | multifocal |  |
| 209 | 15 | f | *ETV6* (NM_01987.4) exon 4 - *NTRK3* (NM_002530.3) exon 1 | *ETV6-NTRK3* | Ricarte-Filho et al.^14^ | nd | Warthin-like PTC | 3.1 | no | metastatic |  |
| 219 | 34 | m | *ETV6* (NM_01987.4) exon 4 - *NTRK3* (NM_002530.3) exon 14 | *ETV6-NTRK3* | Ricarte-Filho et al.^14^ | nd | IEFVPTC | 0.7 | focal | multifocal;  FND and simultaneous FA |  |
| 240 | 24 | f | *ETV6* (NM_01987.4) exon 4 - *NTRK3* (NM_002530.3) exon 14 | *ETV6-NTRK3* | Ricarte-Filho et al.^14^ | nd | IEFVPTC | 2.3 | no | FND; simultaneous OA |  |
| 397 | 43 | f | *ETV6* (NM_01987.4) exon 4 - *NTRK3* (NM_002530.3) exon 1 | *ETV6-NTRK3* | Ricarte-Filho et al.^14^ | nd | IEFVPTC | 2.5 | focal |  |  |
| 383 | 51 | f | *ETV6* (NM_001987.4) exon 4 - *NTRK3* (NM_002530.3) exon 14 | *ETV6-NTRK3* | Ricarte-Filho et al.^14^ | nd | IEFVPTC | 2.4 | + | FND; radiation exposure |  |
| 265 | 60 | m | *ETV6* (NM_001987.4) exon 5 - *NTRK3* exon 13 (NM_001007156.2) | *ETV6-NTRK3* | Huang et al.^16^ | *DDX3X* c.1420G>C, p.Asp474His,  *CREBBP* c.5332T>A, p.Ser1778Thr,  *NTRK3* c.1867_1868delGGinsTT, p.Gly623Leu, probable resistance mechanism after *NTRK* inhibitors.  Full loss of *CDKN2A* | SC | 2.5 | no | metastatic |  |
| 399 | 60 | m | *FGFR2* (NM_000141.4) exon 17 - *WARS1* (NM_004184.4) exon 3 | *FGFR2-WARS1* | Novel | nd | mitotically active encapsulated PTC with a predominant follicular growth pattern^6^ | 2,1 | no | *WARS1* expression detected.  Breast cancer NOS:  *DEGS2-WARS1^17^*  SCC of the lung:  *THSD3-WARS1^17,18^*  One year prior a metastatic CRC with neoadjuvant CHT |  |
| 40 | 30 | m | *GOLGA5* (NM_005113.3) exon 7 - *RET* (NM_0020630.4) exon 12 | *GOLGA5-RET* | Klugbauer and Rabes^19^ | nd | PTC NOS | 0.3 | + |  |  |
| 248 | 26 | m | *KIAA1217* (NM_019590.4) exon 11 - *RET* (NM_020630.4) exon 8 | *KIAA1217-RET* | Bulanova Pekova et al.^20^ | nd | IEFVPTC | 1.1 | no | St.p. RT CML;  simultaneous FA |  |
| 12 | 30 | f | *MKRN1* (NM_013446.3) exon 3 - *BRAF* (NM_004333.4) exon 10 | *MKRN1-BRAF* | Stransky et al.^21^ | not tested | classic PTC | 2.5 | + | multifocal; metastatic |  |
| 5 | 14 | m | *NCOA4* (NM_005437.3) exon 6 - *RET* (NM_020630.4) exon 12 | *NCOA4-RET* | This study* | nd | IEFVPTC | 2.5 | no | metastatic |  |
| 7 | 16 | f | *NCOA4* (NM_005437.3) exon 6 - *RET* (NM_020630.4) exon 12 | *NCOA4-RET* | This study* | nd | oncocytic PTC | 0.7 | + | multifocal; metastatic |  |
| 52 | 66 | f | *NCOA4* (NM_005437.3) exon 7 - *RET* (NM_020630.4) exon 12 | *NCOA4-RET* | Santoro et al.^22^ | not tested | HGDTC | 2.6 | no | atrophic parenchyma |  |
| 63 | 24 | m | *NCOA4* (NM_005437.3) exon 7 - *RET* (NM_020630.4) exon 12 | *NCOA4-RET* | Santoro et al.^22^ | nd | classic PTC | 1.2 | no | metastatic |  |
| 216 | 31 | f | *NCOA4* (NM_005437.3) exon 7 - *RET* (NM_020630.4) exon 12 | *NCOA4-RET* | Santoro et al.^22^ | nd | classic PTC | 0.9 | focal | metastatic |  |
| 218 | 37 | m | *NCOA4* (NM_005437.3) exon 7 - *RET* (NM_020630.4) exon 12 | *NCOA4-RET* | Santoro et al.^22^ | nd | IEFVPTC | 3.5 | focal | multifocal; metastatic |  |
| 282 | 29 | f | *NCOA4* (NM_005437.3) exon 7 - *RET* (NM_020630.4) exon 12 | *NCOA4-RET* | Santoro et al.^22^ | nd | IEFVPTC | 4 | + | multifocal; metastatic |  |
| 376 | 8 | m | *NCOA4* (NM_005437.3) exon 7 - *RET* (NM_020630.4) exon 12 | *NCOA4-RET* | Santoro et al.^22^ | VUS *RET* c.1573C>G, p.(Arg525Gly) | IEFVPTC | 1.1 | no |  |  |
| 379 | 55 | f | *NCOA4* (NM_005437.3) exon 7 - *RET* (NM_020630.4) exon 12 | *NCOA4-RET* | Santoro et al.^22^ | not tested | IEFVPTC | 4.5 | + | metastatic |  |
| 410 | 42 | m | *NCOA4* (NM_005437.3) exon 7 - *RET* (NM_020630.4) exon 12 | *NCOA4-RET* | Santoro et al.^22^ | nd | PTC NOS | 2.5 | no | multifocal; metastatic |  |
| 94 | 46 | f | *NSD3* (NM_017778.2) exon 8 *- NUTM1* (NM_175741.2) exon 2 | *NSD3-NUTM1* | Agaimy et al. ^23^ | nd | SMECE | 2.5 | + |  |  |
| 10 | 17 | f | *PAX8* (NM_00003466.3) exon 8 - *PPARɣ* (NM_005037.54) exon 2 | *PAX8-PPARɣ* | Kroll et al.^24^ | Amplification of *FGFR3* and *FOXL2*, gain of *SMO* | widely invasive FTC | 3.8 | no |  |  |
| 54 | 58 | f | *PAX8* (NM_00003466.3) exon 8 - *PPARɣ* (NM_005037.54) exon 2 | *PAX8-PPARɣ* | Kroll et al.^24^ | nd | IEFVPTC | 2.4 | + |  |  |
| 60 | 41 | f | *PAX8* (NM_00003466.3) exon 8 - *PPARɣ* (NM_005037.54) exon 2 | *PAX8-PPARɣ* | Kroll et al.^24^ | *NRAS* c.181C>A, p.(Gln61Lys) | IEFVPTC | 5.7 | focal | FND; multifocal;  metachronous contralateral IEFVPTC with different fusion |  |
| 73 | 19 | m | *PAX8* (NM_00003466.3) exon 8 - *PPARɣ* (NM_005037.54) exon 2 | *PAX8-PPARɣ* | Kroll et al.^24^ | *APC* c.3920T>A, p.(Ile1307Lys). | minimally invasive FTC | 2.5 | + |  |  |
| 75 | | 29 | f | *PAX8* (NM_00003466.3) exon 8 - *PPARɣ* (NM_005037.54) exon 2 | *PAX8-PPARɣ* | Kroll et al.^24^ | nd | minimally invasive FTC | 5 | + |  |
| 277 | | 40 | f | *PAX8* (NM_00003466.3) exon 8 - *PPARɣ* (NM_005037.54) exon 2 | *PAX8-PPARɣ* | Kroll et al.^24^ | nd | FC NOS | 4.5 | no | metastatic |
| 153 | 69 | f | *PAX8* (NM_00003466.3) exon 8 - *PPARɣ* (NM_005037.54) exon 3 | *PAX8-PPARɣ* | Kroll et al.^24^ | nd | IEFVPTC | 3.3 | focal |  |  |
| 82 | | 47 | f | *PAX8* (NM_00003466.3) exon 9 - *PPARɣ* (NM_005037.54) exon 2 | *PAX8-PPARɣ* | Kroll et al.^24^ | nd | IEFVPTC | 2 | + | FND; simultaneous OA |
| 103 | 41 | m | *PAX8* (NM_00003466.3) exon 10 - *PPARɣ* (NM_005037.54) exon 2 | *PAX8-PPARɣ* | Kroll et al.^24^ | nd | minimally invasive FTC | 8.5 | no | history of low-grade follicular lymphoma |  |
| 232 | 26 | f | *PAX8* (NM_00003466.3) exon 10 - *PPARɣ* (NM_005037.54) exon 2 | *PAX8-PPARɣ* | Kroll et al.^24^ | nd | minimally invasive FTC | 1.8 | + |  |  |
| 134 | 27 | f | *PPP1R21* (NM_024188.2) exon 7 - *ALK* (NM_004304.4) exon 20 | *PPP1R21-ALK* | Panebianco et al.^25^ | nd | IEFVPTC | 2.5 | focal |  |  |
| 281 | 25 | f | *PPP1R21* (NM_024188.2) exon 7 - *ALK* (NM_004304.4) exon 20 | *PPP1R21-ALK* | Panebianco et al.^25^ | nd | IEFVPTC | 2.9 | no |  |  |
| 405 | 79 | m | *PVT1* (NR_003367.3) exon 1 - *MYC* (NM_002467.6) exon 2 | *PVT1-MYC* | L’Abbate et al. in CRC^26^ and Northcott et al. in medulloblastoma ^27^ | *STK11* c.97G>T, p.(Glu33*), *PTEN* c.860C>G, p.(Ser287*) | ATC | na | na |  |  |
| 171 | 47 | f | *SASH1* (NM_015278.3) exon 2 - *BRAF* (NM_004333.4) exon 11 | *SASH1-BRAF* | Aydemirli and Morreau^2^ | nd | classic PTC | 0.15 | + | Two simultaneous PTC’s (*BRAF*^V600E^, pathogenic *BRAF* indel), and FA |  |
| 60 | 41 | f | *SCD5* (NM_024906.2) exon 1 *- MET* (NM_000245.2) exon 2 | *SCD5-MET* | Aydemirli and Morreau^2^ | nd | IEFVPTC | 1.5 | focal | FND; multifocal;  metachronous contralateral IEFVPTC with different fusion |  |
| 362 | 80 | f | *SLC12A7* (NM_006598.2) exon 1 - *TERT* (NM_198253.2) exon 2 | *SLC12A7-TERT* | Robinson et al. in hepatocellular carcinoma ^28^ | *KRAS* c.35G>T, p.(Gly12Val)  Imbalances with copy number gain on chr. 1q, 3, 4, 5, 6, 8, 9, 10, 11, 12, 13, 14, 15, 16, 18, 19, 20 | OCA | 7 | no | metastatic; FND |  |
| 15 | 14 | f | *SPECC1L* (NM_015330.4) exon 10 - *RET*(NM_020630.4) exon 12 | *SPECC1L-RET* | Stransky et al.^21^ | not tested | PTC NOS | 4.2 | + | metastatic |  |
| 359 | 40 | f | *TG* (NM_003235) exon 41 - *DPRX* (XM_011527011.3) exon 1 | *TG-DPRX* | Schmidt et al.^29^ | nd | minimally invasive FTC | 4.8 | no | simultaneous FA or NIFTP |  |
| 223 | | 55 | f | *THADA* (NM_022065.4) exon 36 - *IGF2BP3* (NM_006547.2) exon 1 | *THADA-IGF2BP3* | Yoshihara et al.^30^ | nd | minimally invasive FTC | 3.5 | + |  |
| 108 | 34 | f | *TNIP1* (NM_006058.4) exon 13 – *RET* (NM_020630.4) exon 12 | *TNIP1-RET* | Rogounovitch et al. ^31^ | nd | classic PTC | 6 | focal | metastatic |  |
| 4 | 43 | f | *TPM3* (NM_152263.3) exon 8 - *NTRK1* (NM_002529.3) exon 12 | *TPM3-NTRK1* | Butti et al.^32^ | nd | IEFVPTC | 3 | no | metastatic |  |
| 313 | 37 | f | *TPR* (NM_003292.2) exon 21 - *NTRK1* (NM_002529.3) exon 10 | *TPR-NTRK1* | Greco et al.^33^ | nd | classic PTC | 0.6 | no | simultaneous FTUMP and FA or hyperplastic nodule |  |
| 8 | 12 | f | *TRIM33* (NM_015906.3) exon 16 - *RET* (NM_020630.4) exon 12 | *TRIM33-RET* | Klugbauer and Rabes.^34^ | nd | IEFVPTC | 4 | no | metastatic |  |
| 110 | 22 | m | *TRIM65* (NM_173547.4) exon 5 - *RET* (NM_020630.5) exon 12 | *TRIM65-RET* | Novel | nd | classic PTC | 1,4 | no | *RET* expression.  Simultaneous occult PTC and two FAs |  |
| 17 | 16 | f | *…. -RET* (detected breakpoint via FISH)  RET/PTC1 and RET/PTC3 RT-PCR negative. | *…. -RET* | na | nd | diffuse sclerosing PTC | 9 | + | metastatic |  |
| *unusual breakpoint  CLT chronic lymphocytic thyroiditis, FA follicular adenoma, FND thyroid follicular nodular disease, FTC follicular thyroid carcinoma, FTUMP follicular tumor of unknown malignant potential, FVPTC follicular variant of papillary thyroid carcinoma, IEFVPTC invasive encapsulated follicular variant of papillary thyroid carcinoma, HGDTC high grade differentiated thyroid carcinoma, na not available, nd not detected, NIFTP noninvasive follicular thyroid neoplasm with papillary-like nuclear features, NOS not other specified, PancAC pancreatic adenocarcinoma, OCA oncocytic carcinoma, PTC papillary thyroid carcinoma, SC secretory carcinoma (previously mammary analogue secretory carcinoma of the thyroid), SMECE sclerosing mucoepidermoid carcinoma with eosinophilia, VAF variant allele frequency, VUS variant of unknown significance/pathogenicity | | | | | | | | | | |  |

**References**

1. Marques AR, Espadinha C, Catarino AL, et al. Expression of PAX8-PPAR gamma 1 rearrangements in both follicular thyroid carcinomas and adenomas. J Clin Endocrinol Metab 2002;87(8):3947-52, doi:10.1210/jcem.87.8.8756

2. Aydemirli MD, Morreau H. Multi-UniFocality (MUF), in contrast to multifocality, in thyroid lesions: Relation to lymphocytic thyroiditis. Pathol Int 2024;74(5):274-284, doi:10.1111/pin.13421

3. Nikiforova MN, Nikitski AV, Panebianco F, et al. GLIS Rearrangement is a Genomic Hallmark of Hyalinizing Trabecular Tumor of the Thyroid Gland. Thyroid 2019;29(2):161-173, doi:10.1089/thy.2018.0791

4. Brandler TC, Liu CZ, Cho M, et al. Does Noninvasive Follicular Thyroid Neoplasm With Papillary-Like Nuclear Features (NIFTP) Have a Unique Molecular Profile? Am J Clin Pathol 2018;150(5):451-460, doi:10.1093/ajcp/aqy075

5. Poulikakos PI, Persaud Y, Janakiraman M, et al. RAF inhibitor resistance is mediated by dimerization of aberrantly spliced BRAF(V600E). Nature 2011;480(7377):387-90, doi:10.1038/nature10662

6. WHO Classification of Tumours Editorial Board. Endocrine and neuroendocrine tumours [Internet]. Lyon (France): International Agency for Research on Cancer; 2022 [cited 2025 11 28]. (WHO classification of tumours series, 5th ed.; vol. 10). Available from: <https://tumourclassification.iarc.who.int/chapters/53>.

7. Heinrich K, Fischer LE, De Toni EN, et al. Case of a Patient With Pancreatic Cancer With Sporadic Microsatellite Instability Associated With a BRAF Fusion Achieving Excellent Response to Immunotherapy. JCO Precis Oncol 2023;7(e2200650, doi:10.1200/po.22.00650

8. Hang JF, Chen JY, Kuo PC, et al. A Shift in Molecular Drivers of Papillary Thyroid Carcinoma Following the 2017 World Health Organization Classification: Characterization of 554 Consecutive Tumors With Emphasis on BRAF-Negative Cases. Mod Pathol 2023;36(9):100242, doi:10.1016/j.modpat.2023.100242

9. Fusco A, Grieco M, Santoro M, et al. A new oncogene in human thyroid papillary carcinomas and their lymph-nodal metastases. Nature 1987;328(6126):170-2, doi:10.1038/328170a0

10. Ritterhouse LL, Wirth LJ, Randolph GW, et al. ROS1 Rearrangement in Thyroid Cancer. Thyroid 2016;26(6):794-7, doi:10.1089/thy.2016.0101

11. Demeure MJ, Aziz M, Rosenberg R, et al. Whole-genome sequencing of an aggressive BRAF wild-type papillary thyroid cancer identified EML4-ALK translocation as a therapeutic target. World J Surg 2014;38(6):1296-305, doi:10.1007/s00268-014-2485-3

12. Liang J, Cai W, Feng D, et al. Genetic landscape of papillary thyroid carcinoma in the Chinese population. J Pathol 2018;244(2):215-226, doi:10.1002/path.5005

13. Nakata T, Kitamura Y, Shimizu K, et al. Fusion of a novel gene, ELKS, to RET due to translocation t(10;12)(q11;p13) in a papillary thyroid carcinoma. Genes Chromosomes Cancer 1999;25(2):97-103, doi:10.1002/(sici)1098-2264(199906)25:2<97::aid-gcc4>3.0.co;2-l

14. Ricarte-Filho JC, Li S, Garcia-Rendueles ME, et al. Identification of kinase fusion oncogenes in post-Chernobyl radiation-induced thyroid cancers. J Clin Invest 2013;123(11):4935-44, doi:10.1172/jci69766

15. Seethala RR, Chiosea SI, Liu CZ, et al. Clinical and Morphologic Features of ETV6-NTRK3 Translocated Papillary Thyroid Carcinoma in an Adult Population Without Radiation Exposure. Am J Surg Pathol 2017;41(4):446-457, doi:10.1097/pas.0000000000000814

16. Huang NS, Cao YM, Lu ZW, et al. Mammary analog secretory carcinoma of the thyroid gland: A rare cancer harboring TRK fusion. Oral Oncol 2021;115(105092, doi:10.1016/j.oraloncology.2020.105092

17. Gao Q, Liang WW, Foltz SM, et al. Driver Fusions and Their Implications in the Development and Treatment of Human Cancers. Cell Rep 2018;23(1):227-238.e3, doi:10.1016/j.celrep.2018.03.050

18. Hu X, Wang Q, Tang M, et al. TumorFusions: an integrative resource for cancer-associated transcript fusions. Nucleic Acids Res 2018;46(D1):D1144-d1149, doi:10.1093/nar/gkx1018

19. Klugbauer S, Demidchik EP, Lengfelder E, et al. Detection of a novel type of RET rearrangement (PTC5) in thyroid carcinomas after Chernobyl and analysis of the involved RET-fused gene RFG5. Cancer Res 1998;58(2):198-203

20. Bulanova Pekova B, Sykorova V, Mastnikova K, et al. RET fusion genes in pediatric and adult thyroid carcinomas: cohort characteristics and prognosis. Endocr Relat Cancer 2023;30(12), doi:10.1530/erc-23-0117

21. Stransky N, Cerami E, Schalm S, et al. The landscape of kinase fusions in cancer. Nat Commun 2014;5(1):4846, doi:10.1038/ncomms5846

22. Santoro M, Dathan N, Berlingieri M, et al. Molecular characterization of RET/PTC3; a novel rearranged version of the RETproto-oncogene in a human thyroid papillary carcinoma. Oncogene 1994;9(2):509-516

23. Agaimy A, Tögel L, Stoehr R, et al. NSD3-NUTM1-rearranged carcinoma of the median neck/thyroid bed developing after recent thyroidectomy for sclerosing mucoepidermoid carcinoma with eosinophilia: report of an extraordinary case. Virchows Arch 2021;479(6):1095-1099, doi:10.1007/s00428-021-03103-8

24. Kroll TG, Sarraf P, Pecciarini L, et al. PAX8-PPARgamma1 fusion oncogene in human thyroid carcinoma [corrected]. Science 2000;289(5483):1357-60, doi:10.1126/science.289.5483.1357

25. Panebianco F, Nikitski AV, Nikiforova MN, et al. Characterization of thyroid cancer driven by known and novel ALK fusions. Endocr Relat Cancer 2019;26(11):803-814, doi:10.1530/erc-19-0325

26. L'Abbate A, Macchia G, D'Addabbo P, et al. Genomic organization and evolution of double minutes/homogeneously staining regions with MYC amplification in human cancer. Nucleic Acids Res 2014;42(14):9131-45, doi:10.1093/nar/gku590

27. Northcott PA, Shih DJ, Peacock J, et al. Subgroup-specific structural variation across 1,000 medulloblastoma genomes. Nature 2012;488(7409):49-56, doi:10.1038/nature11327

28. Robinson DR, Wu YM, Lonigro RJ, et al. Integrative clinical genomics of metastatic cancer. Nature 2017;548(7667):297-303, doi:10.1038/nature23306

29. Schmidt GM, Fornal IJ, Doerfler WR, et al. Spectrum and carcinogenic properties of thyroglobulin gene fusions in thyroid. Endocr Relat Cancer 2025, doi:10.1530/erc-24-0334

30. Yoshihara K, Wang Q, Torres-Garcia W, et al. The landscape and therapeutic relevance of cancer-associated transcript fusions. Oncogene 2015;34(37):4845-54, doi:10.1038/onc.2014.406

31. Rogounovitch TI, Mankovskaya SV, Fridman MV, et al. Major Oncogenic Drivers and Their Clinicopathological Correlations in Sporadic Childhood Papillary Thyroid Carcinoma in Belarus. Cancers (Basel) 2021;13(13), doi:10.3390/cancers13133374

32. Butti MG, Bongarzone I, Ferraresi G, et al. A sequence analysis of the genomic regions involved in the rearrangements between TPM3 and NTRK1 genes producing TRK oncogenes in papillary thyroid carcinomas. Genomics 1995;28(1):15-24, doi:10.1006/geno.1995.1100

33. Greco A, Pierotti MA, Bongarzone I, et al. TRK-T1 is a novel oncogene formed by the fusion of TPR and TRK genes in human papillary thyroid carcinomas. Oncogene 1992;7(2):237-42

34. Klugbauer S, Rabes HM. The transcription coactivator HTIF1 and a related protein are fused to the RET receptor tyrosine kinase in childhood papillary thyroid carcinomas. Oncogene 1999;18(30):4388-93, doi:10.1038/sj.onc.1202824
