# Supplementary material for: The Occurrence of Gene Fusions in Thyroid Lesions and the Relation With Chronic Lymphocytic Thyroiditis
Source: Pathol Int. 2026 Jan 5;76(1):e70081. doi: 10.1111/pin.70081 (PMC12835965; doi:10.1111/pin.70081)
Supplement: Supplementary file 3 — Supplemental Material. [file PIN-76-0-s003.docx]

***Previously utilized diverse molecular tests inclusive NGS panels (***For the currently applied molecular test also included in this study please refer to <https://www.palga.nl/voor-pathologen/moleculaire-bepaling> under LUMC)***:***

### **Hotspot mutation analysis (allele specific PCR Taqman):**

*KRAS exon 1 codon 12/13* *detects c.34G>A (p.G12S), c.34G>C (p.G12R), c.34G>T (p.G12C), c.35G>A (p.G12D), c.35G>C (p.G12A), c.35G>T (p.G12V), c.38G>A (p.G13D), c.37G>T (p.G13C)*

*BRAF exon 15** ** detects c.1799T>A (p.V600E) and c.1798GT>AA (p.V600K).*

*EGFR exon 21, c.2573T>G and c.2573_2574TG>GT (p.L858R)*

*EGFR exon 19*

*PIK3CA exon 9 and 20*** *** detects c.1624G>A (p.E542K), c.1633G>A (p.E545K), c.3140A>G (p.H1047R).*

#### **OPTIONAL with Taqman:**

*NRAS exon 1 and exon 2**** **** detects c.35G>A (p.G12D), c.181C>A (Q61K), c.182A>T (p.Q61L),c.182A>G (p.Q61R), c.183A>T (p.Q61H) and/or codons 117, 146.*

*HRAS exon 2***** ***** detects c.181C>A (p.Q61K), c.182A>G (p.Q61R) and/or codon 12/13.*

*KRAS exon 3 (codon 61) and exon 4 (codon 117, 146).*

*P53 exons 5, 6, 7 and 8.*

*BRAF expn 15* c.1799_1800TG>AT / c.1799_1800TG>AC: p.(Val600Asp); c.1798_1799GT>AG: (Val600Arg); c.1798G>A: p.(Val600Met).

### **Sanger sequencing for TERT**

This Sanger sequencing reaction analyzes the region in the TERT promoter containing the variants: NM_198253.2: c.-57A>C (A161C; COSM1717366); c.-124C>T (C228T; COSM1716558); and c.-146C>T (C250T; COSM1716559). Sanger sequencing (of purified PCR products) is performed by a reference laboratory (Macrogen).

### **Somatic mutation analysis Next Generation sequencing (Ampliseq Cancer Hotspot Panel v2):**

NGS Assay characteristics. Following genes are analyzed on Ion Torrent PGM/Proton:

*KRAS* (exon 2-4); *NRAS* (exon 2-4); *HRAS* (exon 2-3); *BRAF* (exon 11,15); *EGFR* (exon 3,7,15,18-21); *GNAQ* (exon 5); *GNAS* (exon 8-9); *IDH1* (exon 4); *IDH2* (exon 4); *KIT* (exon 2, 9-18); *PDGFRA* (exon 12,14,15,18,23); *PIK3CA* (exon 2,5,6-10,14,18,21); *RET* (exon 10-12,15,16); *TP53* (exon 4-8,11).

Hotspots in following genes are analyzed:

*ABL1; AKT1; ALK; APC; ATM; CDH1; CDKN2A; CSF1R; CTNNB1; ERBB2; ERBB4; EZH2; FBXW7; FGFR1; FGFR2; FGFR3; FLT3; GNA11; HNF1A; JAK2; JAK3; KDR; MET; MLH1; MPL; NOTCH1; NPM1; PTEN; PTPN11; RB1; SMAD4; SMARCB1; SMO; SRC; STK11; VHL*

Unless otherwise noted, all sequences have a depth of more than 100 reads, and variants with an allele frequency of 0.1 or greater are reported. Class 1 through 3 variants are not reported. Class 4 is a potentially pathogenic variation, and class 5 is a pathogenic variation. *: stop codon.

### **Somatic mutation analysis Next Generation sequencing (Ampliseq Cancer Hotspot Panel v3):**

NGS Assay characteristics. Following genes are analyzed on Ion Torrent PGM/Proton:
ARAF (7,10); CTNNB1 (1,2,4,7,8,12,15); KRAS (2-4); NRAS (2-4); HRAS (2-3); BRAF (11,15); EGFR (3,7,15,18-21); GNAQ (5); GNAS (8-9); H3F3A (2); H3F3B (2); IDH1 (4); IDH2 (4); KIT (2,9-18); MYD88 (3b,5), MUTYH (7,13); PDGFRA (12,14,15,18,23); PIK3CA (2,5,6-10,14,18,21); POLE (9,11,13,14); RET (10-12,15,16); TP53 (1-11).
Hotspots in following genes are analyzed:
ABL1; AKT1; ALK; APC; ATM; CARD11; CD79A; CD79B; CDH1; CDKN2A; CSF1R; CTNNB1; ERBB2; ERBB4; EZH2; FBXW7; FGFR1; FGFR2; FGFR3; FLT3; GNA11; HNF1A; JAK2; JAK3; KDR; MET; MLH1; MPL; NOTCH1; NPM1; PTEN; PTPN11; RB1; SMAD4; SMARCB1; SMO; SRC; STK11; VHL.
Amplification/Gain and deletions or LOH are examined with an experimental CNV analysis tool. If the tumor cell percentage is less than 40%, the analysis is less reliable. Unless otherwise noted, all sequences have a depth of more than 100 reads, and variants with an allele frequency of 0.1 or greater are reported. Class 1 through 3 variants are not reported. Class 4 is a potentially pathogenic variation, and class 5 is a pathogenic variation. *: stop codon.

**Somatic mutation analysis Next Generation sequencing (Custom Ampliseq Cancer Hotspot Panel v4):**
NGS Assay characteristics. Following genes (exons in brackets) are analyzed on Ion S5:
ARAF (2,4-6,7,9,10,11,15,16); CD79B (5,6); CIC (5); CTNNB1 (1,2,4,7,8,12,15); EIF1AX (1,3-6); ERBB3 (23); KRAS (2-4); NRAS (2-4); HRAS (2-3); BRAF (6,11,15); EGFR (3,7,15,18-21); GNAQ (4,5); GNAS (8-9); H3F3A (2); H3F3B (2); IDH1 (4); IDH2 (4); KIT (2,9-18); MAP2K1 (2-4,6,7,11); MAP2K2 (2,3); MAP2K4 (2,4,5,9); MAP3K1 (5,8,13,14,16,17,20); MDM2 (3,4,6,7,8); MED12 (2); MYD88 (3b,5), MUTYH (7,13); PDGFRA (12,14,15,18,23); PDGFRB (12,14); PIK3CA (2,5,6-10,14,18,21); POLE (9-14); RET (10-12,15,16); TP53 (1-11).
Hotspots in following genes are analyzed:
ABL1; AKT1; ALK; APC; ATM; CARD11; CD79A; CDK4; CDH1; CDKN2A; CSF1R; CTNNB1; ERBB2; ERBB4; EZH2; FBXW7; FGFR1; FGFR2; FGFR3; FLT3; FOXL2; GNA11; HNF1A; JAK2; JAK3; KDR; MET; MLH1; MPL; MYC; NOTCH1; NPM1; PTEN; PTK2; PTPN11; RB1; SMAD4; SMARCB1; SMO; SRC; STK11; VHL.
Amplification/gain and deletions or LOH are examined with a CNV analysis tool. If the tumor cell percentage is less than 40%, the analysis is less reliable. Unless otherwise stated, all sequences have a depth of more than 100 reads, and variants with an allele frequency of 0.05 or greater are reported. Class 1 and 2 variants are not reported. Class 3 are variants with an unknown effect. Class 4 is a potentially pathogenic variation, and class 5 is a pathogenic variation. *: stop codon.

The effect of reported class 3 variants on protein function is currently unknown based on research in the literature (PubMed) and general or locus-specific databases (Mycancergenome, Alamut Visual, NCBI dbSNP, NCBI ClinVar, COSMIC, Jackson laboratory database, LOVD, MD Anderson, IARC TP53 database).

### **NGS ENDO32 v1**

The custom Ampliseq NGS ENDO32 v1 panel analyzes the coding exons of the following genes: ATP1A1, ATP2B3, ARMC5, CACNA1D, KCNJ5, NF1, TSC1, CDKN1A, CDKN1B, CDKN1C, CDKN2A, CDKN2B, CDKN2C, CDKN2D, CDC73, MEN1, and TP53. Hotspots in the following genes are also analyzed: BRAF, DICER1, EIF1AX, HRAS, KRAS, NRAS, PIK3CA, PTEN, RET, and the TERT promoter. Loss of Heterozygosity (LOH) and Copy Number Variation (CNV) are determined for the panel-relevant genes and/or chromosomal regions. The generation of NGS libraries, quality assurance, and data interpretation are the responsibility of the Pathology Department of the LUMC (ISO15189). Next-Generation Sequencing (NGS) is performed by the referral laboratory GenomeScan BV (ISO17025) on the Ion Torrent S5 Genestudio platform.

### **RCPL**

NGS characteristics (details upon request).

The RCPL NGS panel is an updated custom-made panel, previously called ENDO. The results from this panel will initially be "research grade" and will be reported as such. Results for some specific requests are only provided for a subset of the genes and loci within the panel described below, unless otherwise indicated.

The custom Ampliseq NGS RCPL (Rare Cancer Panel Leiden) analyzes the coding exons of the following genes. ARMC5, ATP1A1, ATP2B3, BAP1, CACNA1D, CDC73, CDH1, CDKN1A, CDKN1B, CDKN1C, CDKN2A, CDKN2B, CDKN2C, CDKN2D, CYSLTR2, EIF1AX, KCNJ5, KIT, KRAS, LZTR1, MBD4, MEN1, MTOR, NF1, NF2, PLCB4, PRKAR1A, PTEN, SMARCB1, SMARCE1, SRSF2, SUFU, TP53, TSC1, TSC2, U2AF1. Additionally, hotspots in the following genes are analyzed: BRAF, CCAT1, CDC27, CDK4, DICER1, GNA11, GNAQ, HRAS, IDH1, IDH2, NRAS, PIK3CA, PTK2, RET, SF3B1, and the TERT promoter.

Loss of Heterozygosity (LOH) and Copy Number Variation (CNV) are determined for the panel-relevant genes and/or chromosomal regions. The generation of NGS libraries, quality assurance, and data interpretation are the responsibility of the KMBP (Royal Dutch Society for Pathology) at the LUMC (ISO15189). Next-Generation Sequencing (NGS) is performed by the referral laboratory GenomeScan BV (ISO17025) on the Ion Torrent S5 Genestudio platform.

### **APC panel**

NGS Assay Characteristics

The custom Ampliseq APC NGS panel consists of a pool of primers that detect all coding exons of APC, except for a 70-base region in exon 14, on an Ion Torrent PGM/Proton. The exonuclease domains of POLE/D1, the Dutch MUTYH hotspot mutations, and the NTHL1 hotspot mutation are also analyzed. Unless otherwise noted, all sequences have a depth of more than 100 reads, and variants with an allele frequency of 0.1 or greater are reported. Class 1 through 3 variants are not reported. Class 4 is a potentially pathogenic variation, and class 5 is a pathogenic variation. A * indicates a stop codon.

### **Pre-Archer detection of fusions**

PCR Translocation detection: PAX8/PPARG t(2;3)(q13;p25):

Gene fusion analysis by Next Generation Sequencing of ALK, RET, ROS1 or NTRK1.

NGS Assay Characteristics: The Ampliseq RNA Fusion Lung Cancer Research panel on an Ion Torrent PGM analyzes more than 70 potential gene fusions of ALK, RET, ROS1, and NTRK1. The sensitivity for these fusions is 1%. The analyses are performed in Ion Reporter. Sequences of expression control genes LMNA, TBP, MYC, HMBS, and ITGB7 must be present (>1000 reads). The 3’/5’ imbalance factor in ALK (>=0.025), RET(>=0.045) or ROS1(>=0.5) is an indication for a fusion of the gene with an unknown partner, however an imbalance below this threshold does not rule it out

### **Archer CTL**

#### **V1**

NGS Assay characteristics.

The Archer FusionPlex CTL Panel V1, run on Ion Torrent PGM/Proton, can detect fusions to the following target genes: ALK (exon 5'; 2,4,6,10,16-23, (intron19)), AXL (exon 3';18-20), BRAF (exon 5'; 7-11, exon 3'; 7,8,10), CCND1 (exon 5'; 1-4, exon 3'; 1,2,4), FGFR1 (exon 5'; 2, 8-10,17, exon 3' ; 17), FGFR2 (exon 5'; 2,5,7-10 , exon 3'; 17), FGFR3 (exon 5'; 3,5,8-10, exon 3'; 17, (intron17)), MET (exon 5'; 2,4-6, 13,14,16,17,21, exon 3'; 2), NRG1 (exon 5'; 1,2,3,6), NTRK1 (exon 5'; 2,4,6,8,10-13), NTRK2 (exon 5'; 5,7,9,11-17), NTRK3 (exon 5'; 4,7,10,13-16), PPARG (exon 5'; 1,2,3,5), RAF1 (exon 5'; 4-7, 9-12), RET (exon 5'; 2,4,6,8,9-14), ROS1 (exon 5'; 2,4,7,31-37), THADA (exon 3'; 24-30, 36,37).

#### Fusionplex data are analyzed in the Archer Analysis software (version 5). Unless otherwise stated, only "strong-evidence" fusions are reported. For reliable analysis, the samples meet the criteria below. If no fusion is found and these criteria are not met, the result is considered less reliable, and fusions cannot be ruled out.

#### Criteria:

#### (1) QC score (Ct) must be less than 30. If Ct is 30-31, the fusionplex is considered less reliable; if Ct is >31, the quality is unsuitable.

#### (2) The number of reads per sample must be at least 1.5M.

#### (3) The percentage of RNA reads must be greater than 40%; below this value, the analysis is considered less reliable. The Fusionplex-CTL can also detect hotspot mutations in BRAF (exons 11, 15), HRAS, NRAS (exons 2 and 3, codons 12, 13, 61), KRAS (exons 2, 3 and 4, codons 12, 13, 61, and 146), and the EGFRvIII variant.

Mutations are analyzed in both DNA and RNA, allowing reliable mutation analysis to be performed even with a low RNA percentage.

#### **V2**

The Archer FusionPlex CTL Panel v2 can detect fusions to the following target genes: ALK, AXL, BRAF, CCND1, FGFR1, FGFR2, FGFR3,GLIS1, GLIS3, MET, NRG1, NTRK1, NTRK2, NTRK3, PPARG, RAF1, RET, ROS1, TERT, THADA. B: Tevens kunnen hotspot mutaties aangetoond worden in AKT1, ALK, BRAF, CTNNB1, DDR2, DICER1, EGFR, EIF1AX, ERBB2, FGFR1, GNAS, HRAS, IDH1, IDH2, KRAS, MAP2K1, NRAS, PIK3CA, RET, and ROS1.

Mutations are analyzed in both DNA and RNA, allowing reliable mutation analysis even with a low RNA percentage. The generation of NGS libraries, quality assurance, and data interpretation falls under the KMBP (Royal Dutch Society for Biomedical Research) responsibility of the Pathology Department of the LUMC (ISO15189). Next-Generation Sequencing (NGS) is performed by the referral laboratory GenomeScan BV (ISO17025) on the Ion Torrent S5 Genestudio platform.
