# Supplementary material for: The Occurrence of Gene Fusions in Thyroid Lesions and the Relation With Chronic Lymphocytic Thyroiditis
Source: Pathol Int. 2026 Jan 5;76(1):e70081. doi: 10.1111/pin.70081 (PMC12835965; doi:10.1111/pin.70081)
Supplement: Supplementary file 4 — Supplemental Text 1. [file PIN-76-0-s001.docx]

**Supplemental Text 1**

Patients of Group 1 (n=86, of which 63 female and 23 male patients) were significantly younger (age range 8 – 80 years; mean 37.9; SD 18.2) than patients of Group 2 (n=140, of which 98 female and 42 male patients; age range 15 – 82 years; mean 51.2; SD 16.8) (p<0.0001). That was also seen when these groups were stratified for gender (p<0.0001 for females and p=0.0006 for males). There was no significant age difference between female and male patients in group 1 (p=0.4), while male patients of group 2 were significantly older than female patients in this group (p=0.02).

PTC patients of Group 1 were significantly younger than PTC patients of Group 2 (p<0.0001). In Group 1 there was no significant age difference in patients with PTC and non-PTC malignancies (p=0.06) with tendency of patients with non-PTC malignancies be slightly older. In Group 2 there was significant age difference, where patients with non-PTC malignancies were significant (p=0.01) older than patients with PTC. In Group 1 there was significant difference in the age of patients with malignant lesions vs benign (B) and low-risk (LR) lesions with patients of B and LR group being significantly older than patients of malignant group (p=0.02, mean age for B+LR of Group 1 49.6 years and for malignant of group 1 36 years). This was not observed within group 2 where no significant difference (p=0.2) was observed between B+LR (mean 47.6 years) and malignant (mean 51.8). Interestingly an inversed age relationship was observed. There was also no significant difference in age between B+LR subgroups of Group 1 and 2 (p=0.7) while there was significant difference between malignant subgroups of group 1 and 2 (p<0.0001) as it was observed for the whole groups.

**CLT and age differences (G1=group 1, G2=group 2)**

| Compared groups | | P |
| --- | --- | --- |
| No CLT G1 M | No CLT G2 M | **<0.0001** |
| No CLT G1 PTC | No CLT G2 PTC | **0.0008** |
| CLT G1 M | CLT G2 M | **0.035** |
| CLT G1 Non-PTC | CLT G2 Non-PTC | **0.0145** |

Patients with malignancies and without CLT were significantly younger in group 1 than in group 2 (p<0.0001). This effect could not be observed for non PTC malignancies in the absence of CLT between the two groups (p=0.32) whereas significant difference in age was seen in PTC patients without CLT between both groups (G1 is younger, p=0.0008).

Comparable with the absence of CLT, the patients with malignancies and CLT were significantly younger in group 1 than in group 2 (p=0.035). Interestingly, in the setting of CLT, there was no significant age difference for PTC patients in Group 1 and 2 (p=0.23, mean group 1 36.1 years, mean group 2 42.6 years, difference between means 6,463 ± 5,320 ) while patients with non-PTC malignancies and CLT were significantly younger with age mean almost double less in group 1 than in group 2 (p=0.0145, mean group 1 36 years, mean group 2 62 years; difference between means 25,20 ± 7,810).
